# Insertion of mNeonGreen into the variable domain of DRP-1 permits visualization of functional endogenous protein

**DOI:** 10.17912/micropub.biology.001588

**Published:** 2025-05-08

**Authors:** Eric J. Lambie, Barbara Conradt

**Affiliations:** 1 Cell and Developmental Biology, University College London, London, England, United Kingdom

## Abstract

We used CRISPR-Cas9 editing of the genomic *drp-1* locus in *C. elegans* to test whether the mitochondrial fission function of DRP-1 was retained following insertion of mNeonGreen into the variable domain. We found that DRP-1 activity remains largely intact despite this large internal insertion. Furthermore, in living cells, the internally tagged protein is readily detectable as discrete puncta associated with mitochondria, which presumably represent prospective mitochondrial scission sites. The internally tagged DRP-1 protein represents a powerful new tool for real time *in vivo* analyses of mitochondrial fission and DRP-1 function.

**Figure 1. Internal tagging with mNeonGreen of C. elegans DRP-1 protein:**
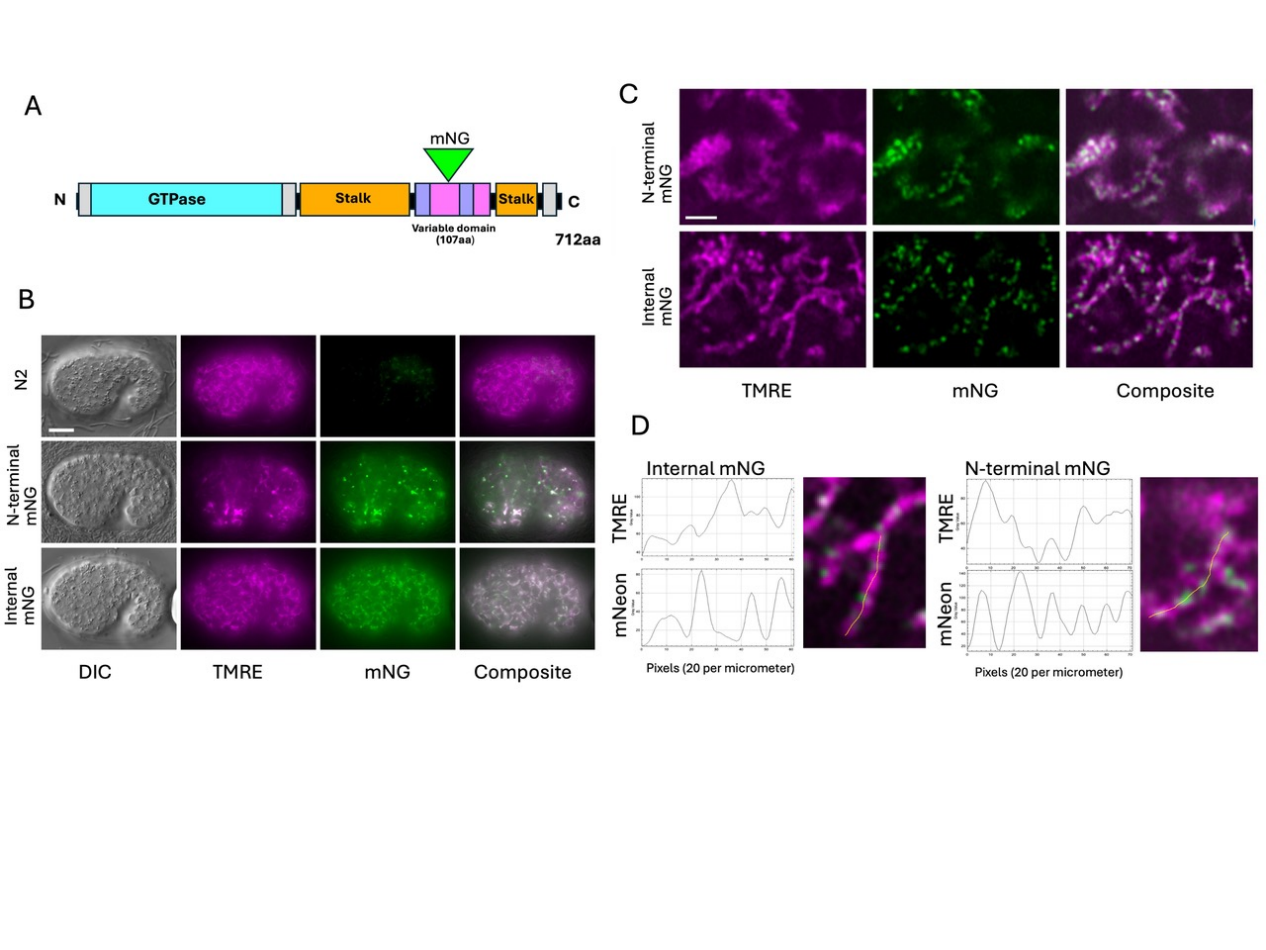
A. Cartoon representation of DRP-1 b isoform showing site of mNG insertion into the variable domain. Gray boxes, BSE elements, lavender boxes, MorF1 and MorF2. In all panels, Internal mNG denotes *drp-1 ( dx230 Internal)* and N-terminal mNG, *drp-1 ( dx224 N-terminal).
* B. Widefield images of TMRE-stained comma stage embryos. Scale bar is 10 micrometers. C. Super-resolution images of TMRE-stained embryos. Scale bar is 2 micrometers. D. Line traces of TMRE and mNG intensity along representative mitochondria from panel C.

## Description

DRP-1 is an evolutionarily conserved, ~80 kDa eukaryotic dynamin-like GTPase that mediates mitochondrial fission (Labrousse et al. 1999; Smirnova et al. 2001; Bleazard et al. 1999; Tábara, Segawa, and Prudent 2025; Kamerkar, Liu, and Higgs 2025). This function depends on the ability of DRP-1 to associate with the mitochondrial outer surface and assemble into helical multimers that cause mitochondrial constriction (Kalia et al. 2018; Rochon et al. 2024). Ultimately, this leads to mitochondrial scission, although the precise role of DRP-1 in the late events of this process is still under investigation (see Roy and Pucadyil 2022; Pérez-Jover et al. 2022). Visualization of functional DRP-1 *in vivo* is confounded by the fact that addition of protein tags to either the N- or C-terminus impairs DRP-1 function (Montecinos-Franjola et al. 2020). We used CRISPR-Cas9-mediated genome editing to test whether insertion of the mNeonGreen (mNG) fluorescent protein into the variable region ( Figure 1 A) is compatible with DRP-1 function. The variable domain is not essential for the assembly of DRP multimers, but it is important for regulation of GTPase activity and proper binding to the outer mitochondrial membrane (Strack and Cribbs 2012; Liu and Chan 2015; Bui et al. 2012). We selected an insertion site that is situated between the conserved MoRF-1 helical region and the cardiolipin-binding domain (Mahajan et al. 2021). This position could potentially allow mNG to fit inside the DRP-1 multimeric complex assembled on the mitochondrial surface. Note that *C. elegans* *drp-1* gives rise to two isoforms, DRP-1 a and DRP-1 b (Sternberg et al. 2024). The b isoform contains 7 additional amino acids (VSAHGEQ) within the variable domain, and these are situated 4aa before the mNG insertion site (see Table 1). Therefore, both isoforms will be tagged with mNG. As a control, we also tested insertion of mNG at the N-terminus of DRP-1 , as this is predicted to cause a loss-of-function phenotype (Montecinos-Franjola et al. 2020). We found that insertion of mNG at the N-terminus ( *drp-1 ( dx224 Nterm)* ) resulted in 39% embryonic lethality (n = 1753). By contrast, insertion of mNG within the variable region ( *drp-1 ( dx230 Internal)* ) resulted in only 9% embryonic lethality (n = 1994). Embryonic lethality in the wild type N2 control strain was 0.35% (n=1409). Neither fusion protein acts as a strong dominant negative, since embryonic lethality was very infrequent among the progeny of heterozygous mothers: Only 0.6 % of the progeny of *drp-1 ( dx230 Internal)* /+ mothers failed to hatch (n = 476) and for *drp-1 ( dx224 Nterm)* /+, this value was 0.5% (n = 570). This also indicates that maternal *drp-1 (+)* rescues the lethality of *drp-1 ( dx224 Nterm)* and *drp-1 ( dx230 Internal)* homozygotes.

Next, we examined the expression and subcellular localization of tagged DRP-1 protein in cleavage stage embryos. For this, we used TMRE (Tetramethylrhodamine ethyl ester) staining to assess colocalization between the mNG-tagged DRP-1 protein and mitochondria ( Figure 1 B). Mitochondrial morphology and distribution during cleavage divisions were very similar between wild type N2 and *drp-1 ( dx230 Internal)* embryos. By contrast, the morphology and distribution of mitochondria in *drp-1 ( dx224 Nterm)* embryos were highly irregular; mitochondria were typically clustered in large aggregates and unequally distributed among blastomeres. This is consistent with a failure in mitochondrial fission. In the case of both *drp-1 ( dx230 Internal)* and *drp-1 ( dx224 Nterm)* , mNG signal was concentrated in foci that colocalized with mitochondria. Super-resolution imaging revealed that both proteins are closely associated with mitochondria ( Figure 1 C). When the TMRE image is used to generate a mask of mitochondrial outlines, 95% of the mNG positive pixels are contained within the masked regions (see methods). DRP-1 ( dx230 Internal) protein is present as discrete puncta ≤200 nm in diameter. These are likely to be ≤ 50 nm oligomeric assemblies of DRP-1 that constrict the mitochondria (Basu et al. 2017; Rosenbloom et al. 2014). Consistent with this idea, the mNG puncta typically coincide with regions of reduced TMRE fluorescence ( Figure 1 D). DRP-1 ( dx224 Nterm) protein tends to associate with mitochondria in the form of both puncta and filamentous aggregates; however, in this case mNG does not correlate with reduced TMRE signal ( Figure 1 D). Finally, DRP-1 ( dx230 Internal) protein appears to be present in a similar subcellular pattern in most if not all cells throughout embryogenesis and larval development as well as in adults.

Overall, our findings suggest that DRP-1 can function relatively normally in a biological context even when mNG is inserted into the variable domain. Therefore, it represents a powerful new tool for real time *in vivo* analyses.

## Methods

Standard methods were used for *C. elegans* culture and genetic manipulations (Brenner 1974), except bacterial strain AMA1004 was used as a food source (Casadaban et al. 1983).

Wild type *C. elegans* N2 (Brenner 1974) was used as the starting strain for CRISPR modifications and for outcrossing edited strains. CRISPR editing was done essentially as described by Paix et al. (Paix et al. 2015), but ssDNA repair templates generated by asymmetric PCR were used on the recommendation of Eroglu et al. (Eroglu, Yu, and Derry 2023). F1 Dpy-10 animals with successful edits were identified by screening for mNG fluorescence using a Nikon SMZ18 epilfuorescence dissecting microscope. The edited *drp-1* alleles were then outcrossed from *dpy-10 (lf)* using N2 .

Reagents for CRISPR-Cas9 editing were obtained from IDT (Coralville, Iowa). Oligonucleotides for PCR were from Merck Life Science (UK). Q5 and LongAmp DNA polymerases were from NEB (UK).

Cas9 crRNA sequences were identified using CRISPOR (http://crispor.gi.ucsc.edu/crispor.py)(Concordet and Haeussler 2018).

Codon optimization and intron placement for mNG were determined using the *C. elegans* codon adapter (https://worm.mpi-cbg.de/codons/cgi-bin/optimize.py) (Redemann et al. 2011)

**Imaging and image processing**

Routine widefield imaging was done using a Zeiss AxioImager M2. Standard DIC optics were used and imaging was done using an EC Plan-Neofluar 100X/1.3 oil objective. For mNG imaging the same objective was used, but with a Colibri 7 LED light source 450-488 nm in combination with filter set 90. Images were acquired with a Photometrics Prime BSI sCMOS camera (USA) using ZEN Blue 2.6 software. For super resolution imaging, we used a Zeiss LSM 980 plus Airyscan 2 detector system. Imaging of mNG was done using 488 nm excitation and > 509 nm emission. Imaging of mScarlet I and TMRE were done separately from mNG using 552 nm excitation and > 578 nm emission. Acquisition parameters were adjusted in ZEN Blue 3.3 as necessary to optimize signal/noise ratio without excessive photobleaching. The standard autofilter setting was used for Airyscan processing.

Mitochondrial localization of DRP-1 ( dx230 Internal) in Figure 1 C was quantified using Fiji as follows. The original pixel value range of the 16 bit images for mNG was 0-203, and for TMRE was 0-216. Images were thresholded by setting the pixel value range to 27-47, saving as 8 bit, then converting to binary mask. Next, Image math was used to subtract the TMRE values from the mNG image. Only 409/8011 filled pixels remained after subtraction, i.e., 94.9% of the signal was removed. When the same subtraction was performed with a TMRE image that was randomized after thresholding, 6662/8011 filled pixels remained, i.e., only 16.7% of the signal was removed.

TMRE staining was done by culturing worms overnight on NGM plates containing 100 nM TMRE (Molecular Probes, Thermo Fisher Scientific).

## Reagents

**Table:** 

Strain	Genotype	Notes		
N2	wild type var. Bristol	From CGC		
EJ1473	drp-1 ( dx230 ) mNG internal	This study		
EJ1480	drp-1 ( dx224 ) mNG N-terminal	This study		
				
DNA molecules	Sequence			
L766 mNeonF	cagtcaaattaaaacaattttcagCAGCTACAACCGggttctggagccggggc	Use with L767 to amplify mNeonGreen from pEL336 to generate repair template for insertion into variable domain of drp-1 .		
L767 mNeonR	GAAGAACAGCGTTCAAGTCGACACCATTAACTCCTGGCCCGCCAGATCCGGCTGA	See above		
Repair template for drp-1 internal	cagtcaaattaaaacaattttcagCAGCTACAACCGggttctggagccggggcttcaggcggtATGCCAGGATCTAAGGGAGAAGAGGATAACATGGCTT CCCTTCCAGCTACTCACGAACTTCATATTTTCGGATCTATCAACGGAGTTGATTTCGATATGGTTGGACAGgtaagtttaaacatatatatactaacta accctgattatttaaattttcagGGAACTGGAAATCCAAACGATGGATACGAAGAGCTTAACCTTAAGTCTACCAAGGGAGATCTTCAATTCTCTCC ATGGATCCTTGTCCCACACATCGGATACGGATTCCACCAATACCTCCCATACCCAGACGGAATGTCCCCATTCCAAGCTGCTATGGTTGATGGA TCTGGATACCAAGTTCACCGCACTATGCAATTCGAGGATGGAGCTTCCCTTACCGTTAACTACCGCTACACTTACGAAGGATCTCACATCAAGG GAGAAGCTCAAGTTAAGGGAACTGGATTCCCAGCTGATGGACCAGTTATGACCAACTCTCTTACCGCTGCTGATTGGTGCCGCTCTAAGgtaa cactatttttgtctctgaaccaactctttaaatttaaatttcagAAGACTTACCCAAACGATAAGACTATCATCTCTACTTTCAAGTGGTCTTACACTACTG GAAACGGAAAGCGCTACCGCTCTACCGCTCGCACTACCTACACCTTCGCTAAGCCAATGGCCGCCAACTACCTCAAGAACCAACCAATGTAC GTCTTCCGTAAGACCGAGCTCAAGCACTCCAAGACCGAGCTCAACTTCAAGGAGTGGCAAAAGGCCTTCACCGACGTCATGGGAATGGACG AGCTCTACAAGGGATCAGGCgccggtgcttcagccggatctggcgggCCAGGAGTTAATGGTGTCGACTTGAACGCTGTTCTTC	Repair template for internal insertion of mNeon. Homology arms in bold, introns lower case.		
L660 mNeonR	ATCCTGCCTAACGTTGCGAAAACATCtTGgAGcTTgTTaACaACgGGAATGAGATTTTCCATcccgccagatccggctga	Use with L722 to generate repair template for insertion of mNeonGreen at 5' end of drp-1 .		
L722 mNeon F	atgatactctcatttcgattcaaaaattttaaattttacagaATGCCAGGATCTAAGGGAGAAGAG	See above		
Repair template N terminal	atgatactctcatttcgattcaaaaattttaaattttacagaATGCCAGGATCTAAGGGAGAAGAGGATAACATGGCTTCCCTTCCAGCTACTCACGAACT TCATATTTTCGGATCTATCAACGGAGTTGATTTCGATATGGTTGGACAGgtaagtttaaacatatatatactaactaaccctgattatttaaattttcagGGA ACTGGAAATCCAAACGATGGATACGAAGAGCTTAACCTTAAGTCTACCAAGGGAGATCTTCAATTCTCTCCATGGATCCTTGTCCCACACATCG GATACGGATTCCACCAATACCTCCCATACCCAGACGGAATGTCCCCATTCCAAGCTGCTATGGTTGATGGATCTGGATACCAAGTTCACCGCAC TATGCAATTCGAGGATGGAGCTTCCCTTACCGTTAACTACCGCTACACTTACGAAGGATCTCACATCAAGGGAGAAGCTCAAGTTAAGGGAAC TGGATTCCCAGCTGATGGACCAGTTATGACCAACTCTCTTACCGCTGCTGATTGGTGCCGCTCTAAGgtaacactatttttgtctctgaaccaactcttt aaatttaaatttcagAAGACTTACCCAAACGATAAGACTATCATCTCTACTTTCAAGTGGTCTTACACTACTGGAAACGGAAAGCGCTACCGCTCT ACCGCTCGCACTACCTACACCTTCGCTAAGCCAATGGCCGCCAACTACCTCAAGAACCAACCAATGTACGTCTTCCGTAAGACCGAGCTCAAG CACTCCAAGACCGAGCTCAACTTCAAGGAGTGGCAAAAGGCCTTCACCGACGTCATGGGAATGGACGAGCTCTACAAGGGATCAGGCgccgg tgcttcagccggatctggcgggATGGAAAATCTCATTCCcGTtGTtAAcAAGCTCCAAGATGTTTTCGCAACGTTAGGCAGGAT	N-terminal repair template. Bolded sequences are flanking homology; synonymous changes introduced to prevent recleavage by Cas9 are indicated by lower case characters. The underlined "ag" sequence is the splice acceptor site for the SL1 trans-spliced leader. Non-bold sequences are mNeon, with introns indicated by lower case lettering.		
pEL336	aaataccgcacagatgcgtaaggagaaaataccgcatcaggcggccttaagggcctcgtgatacgcctatttttataggttaatgtcatgataataatggtttcttagacg tcaggtggcacttttcggggaaatgtgcgcggaacccctatttgtttatttttctaaatacattcaaatatgtatccgctcatgagacaataaccctgataaatgcttcaata atattgaaaaaggaagagtatgagtattcaacatttccgtgtcgcccttattcccttttttgcggcattttgccttcctgtttttgctcacccagaaacgctggtgaaagtaaa agatgctgaagatcagttgggtgcacgagtgggttacatcgaactggatctcaacagcggtaagatccttgagagttttcgccccgaagaacgttttccaatgatgagca cttttaaagttctgctatgtggcgcggtattatcccgtattgacgccgggcaagagcaactcggtcgccgcatacactattctcagaatgacttggttgagtactcaccagt cacagaaaagcatcttacggatggcatgacagtaagagaattatgcagtgctgccataaccatgagtgataacactgcggccaacttacttctgacaacgatcggagga ccgaaggagctaaccgcttttttgcacaacatgggggatcatgtaactcgccttgatcgttgggaaccggagctgaatgaagccataccaaacgacgagcgtgacacca cgatgcctgtagcaatggcaacaacgttgcgcaaactattaactggcgaactacttactctagcttcccggcaacaattaatagactggatggaggcggataaagttgc aggaccacttctgcgctcggcccttccggctggctggtttattgctgataaatctggagccggtgagcgtgggtctcgcggtatcattgcagcactggggccagatggtaa gccctcccgtatcgtagttatctacacgacggggagtcaggcaactatggatgaacgaaatagacagatcgctgagataggtgcctcactgattaagcattggtaactgt cagaccaagtttactcatatatactttagattgatttaaaacttcatttttaatttaaaaggatctaggtgaagatcctttttgataatctcatgaccaaaatcccttaacgt gagttttcgttccactgagcgtcagaccccgtagaaaagatcaaaggatcttcttgagatcctttttttctgcgcgtaatctgctgcttgcaaacaaaaaaaccaccgctac cagcggtggtttgtttgccggatcaagagctaccaactctttttccgaaggtaactggcttcagcagagcgcagataccaaatactgtccttctagtgtagccgtagttagg ccaccacttcaagaactctgtagcaccgcctacatacctcgctctgctaatcctgttaccagtggctgctgccagtggcgataagtcgtgtcttaccgggttggactcaaga cgatagttaccggataaggcgcagcggtcgggctgaacggggggttcgtgcacacagcccagcttggagcgaacgacctacaccgaactgagatacctacagcgtgag cattgagaaagcgccacgcttcccgaagggagaaaggcggacaggtatccggtaagcggcagggtcggaacaggagagcgcacgagggagcttccagggggaaac gcctggtatctttatagtcctgtcgggtttcgccacctctgacttgagcgtcgatttttgtgatgctcgtcaggggggcggagcctatggaaaaacgccagcaacgcggcctt tttacggttcctggccttttgctggccttttgctcacatgttctttcctgcgttatcccctgattctgtggataaccgtattaccgcctttgagtgagctgataccgctcgccgcag ccgaacgaccgagcgcagcgagtcagtgagcgaggaagcggaagagcgcccaatacgcaaaccgcctctccccgcgcgttggccgattcattaatgcagctggcacga caggtttcccgactggaaagcgggcagtgagcgcaacgcaattaatgtgagttagctcactcattaggcaccccaggctttacactttatgcttccggctcgtatgttgtgt ggaattgtgagcggataacaatttcacacaggaaacagctatgaccatgattacgccaagctgtaagtttaaacatgatcttactaactaactattctcatttaaattttc agagcttaaaaatggctgaaatcactcacaacgatggatacgctaacaacttggaaatgaaataagcttgcatgcctgcaggtcgactctagaggatcaccaaaaacg gaacgttgagctggacggaaatagtggtaaagtgacatgattatagtttgaagatttctaatttcacaattagagcaaatgttgttcggtatttattttcaacggtatttata ctattttccacctttttctagaacattcgagctgcttgttgcaaaaggagggcgactcacattcggtacatggaaaagtagtgtacacaataaagagacccagatacattt tccgtctgcgtctctttgcacccaccgggagtattttcaaacgaatgcatctaggaccttctagaacattctgtaaggctgcagaatgcgggtatataaggaaagcgggct cagaggaagccaacacgctttgttctagtgcatctaaaaaacttcgaaaatcctcatcgggatcctatcgattcgcggccgctgtacacccccaaaggacccaaaggtat gtttcgaatgatactaacataacatagaacattttcaggaggacccttggagggtaccgagctcagaaaaaactagttAtgGGTTCCTGTATTGGAAaAATGgCTc ccggttctggagccggggcttcaggcggtATGCCAGGATCTAAGGGAGAAGAGGATAACATGGCTTCCCTTCCAGCTACTCACGAACTTCATATTTTC GGATCTATCAACGGAGTTGATTTCGATATGGTTGGACAGgtaagtttaaacatatatatactaactaaccctgattatttaaattttcagGGAACTGGAAAT CCAAACGATGGATACGAAGAGCTTAACCTTAAGTCTACCAAGGGAGATCTTCAATTCTCTCCATGGATCCTTGTCCCACACATCGGATACGGAT TCCACCAATACCTCCCATACCCAGACGGAATGTCCCCATTCCAAGCTGCTATGGTTGATGGATCTGGATACCAAGTTCACCGCACTATGCAATT CGAGGATGGAGCTTCCCTTACCGTTAACTACCGCTACACTTACGAAGGATCTCACATCAAGGGAGAAGCTCAAGTTAAGGGAACTGGATTCC CAGCTGATGGACCAGTTATGACCAACTCTCTTACCGCTGCTGATTGGTGCCGCTCTAAGgtaacactatttttgtctctgaaccaactctttaaatttaaat ttcagAAGACTTACCCAAACGATAAGACTATCATCTCTACTTTCAAGTGGTCTTACACTACTGGAAACGGAAAGCGCTACCGCTCTACCGCTCGC ACTACCTACACCTTCGCTAAGCCAATGGCCGCCAACTACCTCAAGAACCAACCAATGTACGTCTTCCGTAAGACCGAGCTCAAGCACTCCAAG ACCGAGCTCAACTTCAAGGAGTGGCAAAAGGCCTTCACCGACGTCATGGGAATGGACGAGCTCTACAAGGGATCAGGCgccggtgcttcagccg gatctggcgggCtgatcgacgccaacgtcgttgaattttcaaattttaaatactgaatatttgttttttttcctattatttatttattctctttgtgttttttttcttgctttctaaaa aattaattcaatccaaatctaaacatttttttttctctttccgtctcccaattcgtattccgctcctctcatctgaacacaatgtgcaagtttatttatcttctcgctttcatttcat taggacgtggggggaattggtggaagggggaaacacacaaaaggatgatggaaatgaaataaggacacacaatatgcaacaacattcaattcagaaatatggagg aaggtttaaaagaaaacataaaaatatatagaggaggaaggaaaactagtaaaaaataagcaaagaaattaggcgaacgatgagaattgtcctcgcttgggccctt tcgtctcgcgcgtttcggtgatgacggtgaaaacctctgacacatgcagctcccggagacggtcacagcttgtctgtaagcggatgccgggagcagacaagcccgtcagg gcgcgtcagcgggtgttggcgggtgtcggggctggcttaactatgcggcatcagagcagattgtactgagagtgcaccatatgcggtgtg	Phsp-16::mNeonGreen plasmid used as template for amplification of mNeon Green		
				
Cas9 guide RNAs	Sequence			
DRP1.1	ACACCAUUAACUCCUGGUAC	crRNA for internal insertion of mNeon		
DRP1.2	UCCUGUAGUUUAUUGACGAC	crRNA for N-terminal tagging		
DPY-10	GCUACCAUAGGCACCACGAG	No repair template was used		
				
Fusion protein junctional sequence	Amino acid sequence			
mNeon:: DRP-1 (N-terminal mNeon)	MPGSKGEEDNMASLPATHELHIFGSINGVDFDMVGQGTGNPNDGYEELNLKSTKGDLQFSPWILVPHIGYGFHQYLPYPDGMSPFQAAM VDGSGYQVHRTMQFEDGASLTVNYRYTYEGSHIKGEAQVKGTGFPADGPVMTNSLTAADWCRSKKTYPNDKTIISTFKWSYTTGNGKRYR STARTTYTFAKPMAANYLKNQPMYVFRKTELKHSKTELNFKEWQKAFTDVMGMDELYKGSGAGASAGSGGMENLIP	Underlined sequence is flexible linker. Bold is beginning of DRP-1 .		
		DRP-1 ::mNeon:: DRP-1 (internal mNeon)	VSAHGEQQLQPGSGAGASGGMPGSKGEEDNMASLPATHELHIFGSINGVDFDMVGQGTGNPNDGYEELNLKSTKGDLQFSPWILVPHIGYGFHQYLPYPDGMSPFQAAMVDGSGYQVHRTMQFEDGASLTVNYRYTYEGSHIKGEAQVKGTGFPADGPVMTNSLTAADWCRSKKTYPNDKTIISTFKWSYTTGNGKRYRSTARTTYTFAKPMAANYLKNQPMYVFRKTELKHSKTELNFKEWQKAFTDVMGMDELYKGSGAGASAGSGGPGVNG	DRP-1 flanking residues in bold. Underlined residues are flexible linker sequences. A single valine was deleted from DRP-1 at the junction.
